# Determinants of Intention to Use Mobile Phone Caller Tunes to Promote Voluntary Blood Donation: Cross-Sectional Study

**DOI:** 10.2196/mhealth.9752

**Published:** 2018-05-04

**Authors:** Bernard Appiah, James N Burdine, Ammar Aftab, Lucy Asamoah-Akuoko, David A Anum, Irene A Kretchy, Elfreda W Samman, Patience B Appiah, Imelda Bates

**Affiliations:** ^1^ Research Program on Public and International Engagement for Health Department of Environmental and Occupational Health, Texas A&M School of Public Health Texas A&M University College Station, Texas, TX United States; ^2^ Centre for Science and Health Communication Accra Ghana; ^3^ Department of Health Promotion and Community Health Sciences Texas A&M School of Public Health Texas A&M University College Station, Texas, TX United States; ^4^ Department of Health Policy and Management Texas A&M School of Public Health Texas A&M University College Station, Texas, TX United States; ^5^ Research and Development National Blood Service Ghana Accra Ghana; ^6^ Liverpool School of Tropical Medicine Liverpool United Kingdom; ^7^ Department of Pharmacy Practice and Clinical Pharmacy School of Pharmacy University of Ghana Legon, Accra Ghana

**Keywords:** caller tunes, blood donation, sub-Saharan Africa, technology acceptance model, mobile health

## Abstract

**Background:**

Voluntary blood donation rates are low in sub-Saharan Africa. Sociobehavioral factors such as a belief that donated blood would be used for performing rituals deter people from donating blood. There is a need for culturally appropriate communication interventions to encourage individuals to donate blood. Health care interventions that use mobile phones have increased in developing countries, although many of them focus on SMS text messaging (short message service, SMS). A unique feature of mobile phones that has so far not been used for aiding blood donation is caller tunes. Caller tunes replace the ringing sound heard by a caller to a mobile phone before the called party answers the call. In African countries such as Ghana, instead of the typical ringing sound, a caller may hear a message or song. Despite the popularity of such caller tunes, there is a lack of empirical studies on their potential use for promoting blood donation.

**Objective:**

The aim of this study was to use the technology acceptance model to explore the influence of the factors—perceived ease of use, perceived usefulness, attitude, and free of cost—on intentions of blood or nonblood donors to download blood donation-themed caller tunes to promote blood donation, if available.

**Methods:**

A total of 478 blood donors and 477 nonblood donors were purposively sampled for an interviewer-administered questionnaire survey at blood donation sites in Accra, Ghana. Data were analyzed using descriptive statistics, exploratory factor analysis, and confirmatory factory analysis or structural equation modeling, leading to hypothesis testing to examine factors that determine intention to use caller tunes for blood donation among blood or nonblood donors who use or do not use mobile phone caller tunes.

**Results:**

Perceived usefulness had a significant effect on intention to use caller tunes among blood donors with caller tunes (beta=.293, *P*<.001), blood donors without caller tunes (beta=.165, *P*=.02, nonblood donors with caller tunes (beta=.278, *P*<.001), and nonblood donors without caller tunes (beta=.164, *P*=.01). Attitudes had significant effect on intention to use caller tunes among blood donors without caller tunes (beta=.351, *P*<.001), nonblood donors with caller tunes (beta=.384, *P*<.001), nonblood donors without caller tunes (beta=.539, *P*<.001) but not among blood donors with caller tunes (beta=.056, *P*=.44). The effect of free-of-cost caller tunes on the intention to use for blood donation was statistically significant (beta=.169, *P*<.001) only in the case of nonblood donors without caller tunes, whereas this path was statistically not significant in other models.

**Conclusions:**

Our results provide empirical evidence for designing caller tunes to promote blood donation in Ghana. The study found that making caller tunes free is particularly relevant for nonblood donors with no caller tunes.

## Introduction

### Background

Lack of adequate blood for transfusions is a major global health challenge in low- and middle-income countries, particularly those in sub-Saharan Africa [1,2]. According to the World Health Organization (WHO), countries need to achieve blood donation rates of at least 1 per 100 population [3]. However, of 67 countries worldwide that fall below this target, 38 are in the WHO Africa region [3]. Lack of adequate blood donation in sub-Saharan Africa has been attributed partly to sociocultural beliefs [4-7]. One such belief is that donated blood would be used for performing rituals [8]. These beliefs could be addressed through use of culturally appropriate communication interventions, including the use of face-to-face communication [9-12], mass media [11-16], and mobile phones [10,11,16,17].

According to a 2015 Pew Research Center report, mobile phone ownership has surged in sub-Saharan Africa, with, for example, Ghana and Kenya, respectively, having 83% and 82% of the population owning mobile phones in 2014, up from the 2002 figure of nearly 10% for both countries [18]. The report shows that across seven African countries surveyed, 80% use mobile phones to send SMS text messages (short message service, SMS), but only 14% and 18% get consumer and health information, respectively, through their mobile phones.

Health care interventions that use mobile phones have increased in developing countries [19], although many of them focus on SMS text messages. The use of mobile phone apps for encouraging voluntary blood donation is increasing in the Western countries, but less so in sub-Saharan Africa [20].

In sub-Saharan Africa, some blood transfusion services have used mobile phone SMS text messages to increase awareness about blood donation [17,21]. Although SMS text messages have an important role to play in health care, they do not reach audiences with limited ability to read or write [22]. Thus, there is a need for culturally appropriate mobile health (mHealth) interventions, including use of voice messages created in languages spoken by the target audience.

### Mobile Phone Caller Tunes as New Communication Phenomenon

One popular phenomenon in sub-Saharan Africa is mobile phone caller tunes. A caller tune is the sound a caller to a mobile phone hears before the receiver picks the call [23]. Normally, one hears the “ring, ring, ring” sound. However, in some sub-Saharan African countries, a caller to a mobile phone could hear a song or message in place of the “ring, ring, ring” sound.

Caller tunes operate with a logic reverse to that of ring tones. A ring tone is the sound the called party hears. However, a caller tune, also called ringback tone, is the sound the caller hears [24]. A ring tone is usually available as part of the phone settings. A caller tune, however, is usually determined by the mobile telecommunication operator. The normal “ring, ring ring” sound a caller hears is free. However, mobile phone subscribers who download particular songs or messages as caller tunes may pay a relatively small monthly fee to their mobile phone telecommunication companies [25]. Worldwide, many telecommunication companies have caller tunes. For example, in the United States, T-Mobile has caller tunes. Telecommunication companies such as MTN, Vodafone, and Airtel that operate in Africa and Asia also have caller tunes.

Despite the popularity of such caller tunes, there is lack of empirical studies on its potential use for promoting health or improving health care services.

### Context: Blood Donation Recruitment Strategies in Ghana

In Ghana, blood donor recruitment strategies include visiting schools, workplaces, and places of worship such as churches and mosques to collect blood. During school recesses, blood collection teams struggle to get enough voluntary blood donors [26]. Thus, hospitals have to rely on “family” blood donors. Such blood donors may include others who donate blood for money, but present themselves as family members.

There is a need to attract first time voluntary blood donors and retain them as repeat donors because these are the safest donors. The WHO has a target of requiring all countries to get 100% of all donated blood from voluntary, unpaid donors by 2020 [27]. Thus, the National Blood Service Ghana has been exploring opportunities to increase voluntary blood donation. In a qualitative study to explore how journalists, clinicians, and blood donors could team up to promote blood donation in Ghana, the potential of using mobile phones caller tunes was identified [11].

The overall goal that this project will contribute to is to assess the feasibility of using mobile phone caller tunes on blood donation, if created, to increase the number of blood donors and those who go on to donate regularly.

### Theoretical Foundation

We selected technology acceptance model (TAM) for this study partly because of its use in assessing user acceptance of novel mobile technologies, particularly in health care [28]. Additionally, our primary reason to use TAM was because it has been tested widely in information systems research [29], including its extensions to include new variables [30].

Although we could not identify a single study that had used TAM to evaluate intention to adopt caller tunes to promote blood donation, TAM constructs such as perceived ease of use, perceived usefulness, attitudes to technologies, and intention to use technologies is particularly applicable to this study because of their validation in health information system environments [31].

**Figure 1:**
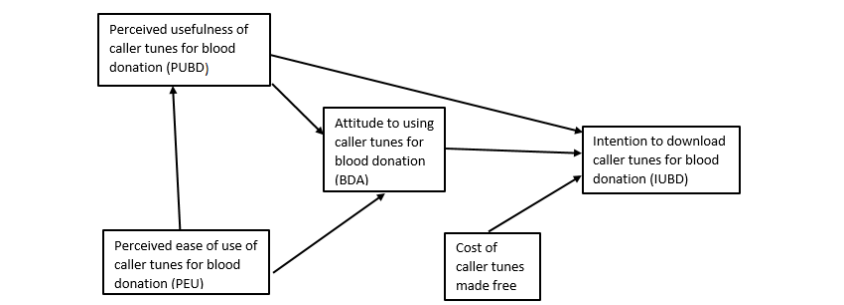
Conceptual research model (technology acceptance model).

TAM posits that perceived ease of use and perceived usefulness of a mobile technology such as caller tune positively influence the attitudes about the technology, with attitudes positively influencing intention to use the technology [32]. Moreover, both perceived usefulness and intention to use the technology positively influence actual use of the technology (Figure 1). We introduced a factor “free of cost” of downloading caller tunes to the original TAM.

### Research Models and Hypotheses

We adapted TAM and used it in the context of mobile phone caller tunes to predict intentions of blood donors and nonblood donors to download caller tunes to promote blood donation. We tested the TAM (as shown in Figure 1) for blood or nonblood donors who use or do not use caller tunes. We focused on these populations because our aim was to explore whether caller tunes could increase the number of blood donors and those who go on to donate regularly.

We introduced “free of cost” of downloading caller tune as a predictor of intention to use caller tunes as seen in Figure 1. The original TAM did not have this variable. However, cost has been operationalized in a prior TAM study as “affordability” or “device perceived as affordable” [33].

### Perceived Ease of Use

Perceived ease of use was defined by Davis as “the degree to which a person believes that using a particular system would be free of effort” [32]. We adapted this definition to the context of mobile phone caller tunes to mean the extent to which users feel it is easy to download caller tunes onto a mobile phone. In general, the belief that it is easy to use a particular health information technology has an influence on one’s intention to use the technology. Many studies have shown that perceived ease of use significantly predicts attitudes to technology [32,34-37] and perceived usefulness of the technology [38-41]. Thus, we test the following hypotheses:

Hypothesis 1: perceived ease of use will have a positive effect on perceived usefulness of caller tunes among blood donors with or without caller tunes.

Hypothesis 2: perceived ease of use will have a positive effect on attitudes to caller tunes among nonblood donors with or without caller tunes.

### Perceived Usefulness

Defined by Davis as “the degree to which a person believes that using a particular system would enhance his or her job performance” [32], perceived usefulness is known to have positive effect on attitudes. We adapted this definition to indicate the extent to which users believe caller tunes could help encourage callers to mobile phones to become blood donors, and thus, increase voluntary blood donation. The belief that a given technology is useful could make people develop attitudes to using it and increase their intention to use it. For example, studies show significant relationship between perceived usefulness and attitudes to a health information [31,40,41] and intention to use technology [31,33,38,42].

Thus, based on these results, our third and fourth hypotheses are as follows:

Hypothesis 3: perceived usefulness will have a positive effect on attitudes to caller tunes among blood donors or nonblood donors with or without caller tunes.

Hypothesis 4: perceived usefulness will have positive effects on intention to use caller tunes among blood donors or nonblood donors with or without caller tunes.

### Attitudes

Attitude was adopted from the theory of reasoned action that posits that intention to perform a given behavior is determined by an individual's overt behavior [43]. Some studies have shown significant relationship between attitudes and behavioral intention [31,40,41]**.** Thus, this study explores the relationship between attitude and behavioral intention in the context of using mobile phone caller tunes for aiding blood donation.

Hypothesis 5: attitudes toward caller tunes will have positive effects on intention to use caller tunes among blood donors or nonblood donors with or without caller tunes.

### Free of Cost

The cost of a technology could determine whether people intend to use it or will actually use it. For example, affordability has been found to influence patients’ intention to use point-of-care medical devices for medical testing [33]. Moreover, cost negatively influences the adoption of mHealth services [44]. Thus, we assessed whether blood donation-themed caller tunes that are to be made free to download could influence the intentions of blood donors and nonblood donors to use them to encourage callers to donate blood.

Hypothesis 6: making caller tunes free to download will have positive effects on intention to download it for promoting blood donation.

### Behavioral Intention

Behavioral intention is a construct that describes people’s target or goal to use a future product or service [45]. Studies show significant relationship between intention and actual use [46,47]. Because caller tunes on blood donation have not yet been developed, this study does not assess the relationship between intention and actual use of mobile phone caller tunes for promoting blood donation. Currently, mobile phone caller tunes in Ghana include songs of popular artistes and religious messages. The outcome of this feasibility study would inform the design and use of caller tunes to promote blood donation among blood or nonblood donors.

## Methods

### Survey Development

Two questionnaires, one for blood donors and another for nonblood donors, were adapted from previous studies on TAM conducted outside Ghana (eg, [48-57], see Multimedia Appendix 1). The questionnaires were then pretested among 5 blood donors and 5 nonblood donors at the Korle Bu Blood Bank in Accra, Ghana for ease and understanding of use. The pretest resulted in reduced overall number of questionnaire items because some respondents found it time-consuming in answering questions on use of caller tunes for promoting blood donation, medication adherence, and patient reporting of adverse drug reactions (ADRs). Seven-point Likert scale with answers ranging from “strongly agree” (1) to “strongly disagree” (7) was used to measure perceived ease of use, perceived usefulness, attitudes, behavioral intention, and free of cost as presented in Multimedia Appendix 1.

### Data Collection

Blood donors and nonblood donors were recruited for the questionnaire survey at blood donation sites such as schools, churches, hospitals, and workplaces in Accra, Ghana. Purposive sampling [58] was used to recruit blood or nonblood donors because it was practically impossible to have a list of all blood donors or nonblood donors at blood donation sites to be used for random sampling. We were also interested in the perspectives of blood or nonblood donors; hence, the use of purposive sampling.

The inclusion criteria for both blood or nonblood donors were being at least 18 years of age and being able to understand English. In addition, blood donors were selected if they indicated that they have donated blood before, including on the day of the interview. The survey did not identify whether those who indicated that they had donated blood before were unpaid blood donors, paid blood donors, or irregular blood donors such as those who donate blood for family members or friends.

Each prospective blood donor or nonblood donor who understood English was provided with information about the study verbally. Those who consented to the study were interviewed. Trained interviewers read the questionnaires in English to the participants and recorded participants’ responses on the paper-based survey questionnaires, which were designed in English (see Multimedia Appendix 2). On average, each interview lasted about 20 min.

The survey questionnaires required participants to answer questions on TAM constructs related to blood donation and intention to use mobile phone caller tunes to promote blood donation, medication adherence, and patient reporting of ADRs as part of a larger project. The focus of this study is on the blood donation component. Data collection occurred from October 2016 to December 2016.

A total of 955 questionnaires were successfully completed for 478 blood donors and 477 nonblood donors out of the 965 who met the inclusion criteria and were approached, representing 99.0% response rate. The 10 agreed to participate but did not complete the questionnaires because they had to leave the blood donation sites. Each respondent received Ghana Cedi equivalent of US $1 as compensation for the time spent in responding to the questionnaires. The study protocol was approved by ethics committees of the Ghana Health Service (GHS-ERC 05/08/16) and Texas A&M University (IRB2016-0655D).

Among the 955 participants, 49.2% (470/955) did not have a mobile phone caller tune, and 50.8% (485/955) had mobile phone caller tune (see Table 1).

**Table 1:** Demographic characteristics of blood or nonblood donors with or without caller tunes.

Characteristic		Total, n (%)
**Gender**		
	Male	633 (66.6)
	Female	317 (33.4)
**Age in years**		
	18-20	365 (38.3)
	21-30	469 (49.2)
	31-40	85(8.9)
	41-50	26(2.7)
	51-60	5(0.5)
	>60	3 (0.3)
**Blood donation status**		
	Nonblood donor	477 (49.9
	Blood donor	478 (50.1)
**Mobile phone has caller tune**		
	No	470 (49.2)
	Yes	485 (50.8)
**Education**		
	Primary	19 (2.0)
	Middle school	11 (1.2)
	Junior high school	68 (7.1)
	Senior high school	345 (36.2)
	Above senior high school	509 (53.5)

### Measurement

Data from the survey were divided into four subsets based on blood donation status and the use of caller tune, namely blood donors with caller tuners, blood donors who do not use caller tunes, nonblood donors with caller tunes, and nonblood donors who did not have caller tunes. The data from each subset (with sample sizes 200-278) were analyzed using descriptive statistics, exploratory factor analysis, and confirmatory factor analysis or structural equation modeling. Results based on non-normality tests showed that each data for a subgroup was not normally distributed as revealed by significant values for Shapiro-Wilk test statistics and high values of kurtosis.

We used TAM for structural equation modeling analysis with the aid of maximum likelihood estimation routines in IBM SPSS AMOS version 25 (IBM Corp). We selected maximum likelihood estimation approach because it performs reasonably well for non-normal data under analytic conditions such as excessive kurtosis and small sample sizes [59], especially those less than 1000 [60].

There were five measures in the model: perceived usefulness, perceived ease of use, attitude, free of cost, and intention to use. Confirmatory factor analysis was conducted for perceived ease of use (seven items for those with caller tunes and three items for those without caller tunes), attitudes to using caller tunes (four items), and perceived usefulness (three items), but not for free of cost and intention to download caller tunes because each had only one item. Multimedia Appendix 1 outlines all items and their corresponding statements. Single items for measuring intention have been used in previous TAM research [61], especially when the aim was to shorten the survey [62].

### Reliability and Validity

Results based on use of IBM SPSS version 24 (IBM Corp) for descriptive analyses of the constructs showed that composite reliability scores were all well above the 0.7 level threshold, and Cronbach alpha scores were also well above .7 threshold [63] for blood donors with caller tunes (see Table 2), nonblood donors with caller tunes (see Table 3), blood donors with no caller tunes (see Table 4), and nonblood donors with caller tunes (see Table 5). We assessed discriminant validity—the extent to which a given construct is different from the others indicated in the instrument—by using the average variance extracted (AVE), which determines the mean variance shared between a given construct and how it was measured. Discriminant validity is established when the AVE is at least 0.50 [63]. Such a figure shows that the construct indicated at least 50% of the measurement variance. Tables 2-5 show AVE scores greater than 0.5 and Cronbach alpha values of at least 0.7, thus suggesting that the instrument used for the study met acceptable validity and reliability levels.

**Table 2:** Factor analysis, reliability, and validity of measures for blood donors with caller tunes. Multimedia Appendix 1 outlines all items and their corresponding statements.

Item		Internal reliability			Convergent validity	
		Cronbach alpha	Item-total correlation	Factor loading	Composite reliability	Average variance extracted
**Perceived ease of use (PEU)**		.893			0.92	0.61
	PEU1		0.63	0.72		
	PEU2		0.74	0.82		
	PEU3		0.74	0.82		
	PEU4		0.67	0.76		
	PEU5		0.70	0.79		
	PEU6		0.74	0.82		
	PEU7		0.64	0.74		
**Perceived usefulness for blood donation (PUBD)**		.862			0.92	0.77
	PUBD1		0.75	0.89		
	PUBD2		0.74	0.89		
	PUBD3		0.74	0.88		
**Attitudes to using caller tunes for blood donation (BDA)**		.851			0.90	0.70
	BDA1		0.74	0.87		
	BDA2		0.65	0.80		
	BDA3		0.77	0.89		
	BDA4		0.63	0.79		

**Table 3:** Factor analysis, reliability, and validity of measures for blood donors with no caller tunes. Multimedia Appendix 1 outlines all items and their corresponding statements.

Item		Internal reliability			Convergent validity	
		Cronbach alpha	Item-total correlation	Factor loading	Composite reliability	Average variance extracted
**Perceived ease of use (PEU)**		.859			0.92	0.78
	PEU1		0.76	0.90		
	PEU2		0.83	0.93		
	PEU3		0.63	0.82		
**Perceived usefulness for blood donation (PUBD)**		.811			0.89	0.73
	PUBD1		0.61	0.82		
	PUBD2		0.76	0.90		
	PUBD3		0.63	0.84		
**Attitudes to using caller tunes for blood donation (BDA)**		.792			0.87	0.63
	BDA1		0.67	0.83		
	BDA2		0.56	0.76		
	BDA3		0.66	0.82		
	BDA4		0.57	0.77		

**Table 4:** Factor analysis, reliability, and validity of measures for nonblood donors with caller tunes. Multimedia Appendix 1 outlines all items and their corresponding statements.

Item		Internal reliability			Convergent validity	
		Cronbach alpha	Item-total correlation	Factor loading	Composite reliability	Average variance extracted
**Perceived ease of use (PEU)**		.84			0.88	0.51
	PEU1		0.63	0.75		
	PEU2		0.58	0.71		
	PEU3		0.58	0.71		
	PEU4		0.44	0.56		
	PEU5		0.58	0.70		
	PEU6		0.65	0.77		
	PEU7		0.63	0.75		
**Perceived usefulness for blood donation (PUBD)**		.84			0.90	0.75
	PUBD1		0.64	0.83		
	PUBD2		0.78	0.91		
	PUBD3		0.68	0.86		
**Attitudes to using caller tunes for blood donation (BDA)**		.82			0.88	0.65
	BDA1		0.65	0.81		
	BDA2		0.60	0.78		
	BDA3		0.65	0.81		
	BDA4		0.66	0.82		

**Table 5:** Factor analysis, reliability, and validity measures for nonblood donors with no caller tunes. Multimedia Appendix 1 outlines all items and their corresponding statements.

Item		Internal reliability			Convergent validity	
		Cronbach alpha	Item-total correlation	Factor loading	Composite reliability	Average variance extracted
**Perceived ease of use (PEU)**		.88			0.93	0.81
	PEU1		0.78	0.91		
	PEU2		0.85	0.94		
	PEU3		0.69	0.85		
**Perceived usefulness for blood donation (PUBD)**		.90			0.94	0.84
	PUBD1		0.72	0.86		
	PUBD2		0.89	0.95		
	PUBD3		0.83	0.93		
**Attitudes to using caller tunes for blood donation (BDA)**		.92			0.95	0.82
	BDA1		0.82	0.90		
	BDA2		0.85	0.92		
	BDA3		0.84	0.91		
	BDA4		0.78	0.87		

## Results

### Descriptive Analysis

The means of the constructs as indicated in Table 6 show that respondents had positive views about using mobile phone caller tunes for promoting blood donation given that the 7-point Likert scale ranged from 1 (strongly agree) to 7 (strongly disagree).

### Model Fit and Structural Models

Three of the research models had goodness of fit indices showing good fit to the data as shown in Table 7. Only the model for nonblood donors with caller tunes had values of normed fit index (NFI), comparative fit index (CFI), and incremental fit index (IFI) not meeting recommended levels [64]. The root mean square error of approximation (RMSEA) value for blood donors with no caller tune was 0 and that for nonblood donors with caller tunes was 0.11, whereas that for blood donors with caller tunes was 0.07 and nonblood donors with no caller tunes was 0.08 (Table 8). At 90% CI, the *P* values of the RMSEA for all but the model involving nonblood donors with caller tunes were not statistically significant (Table 7). All the models met the recommended values of chi square or degree of freedom ratio [65].

### Hypothesis Tests

Most of the results support the proposed hypotheses (Table 8).

**Figure 2:**
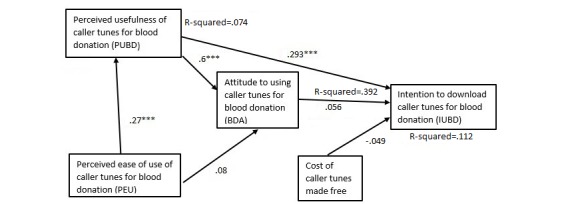
Path model for blood donors with caller tunes. ***P<.001.

**Figure 3:**
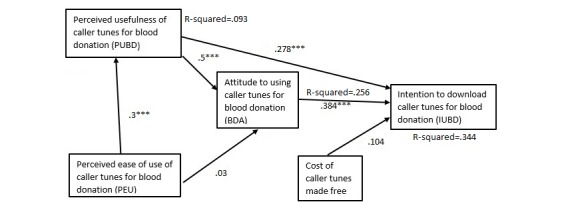
Path model for nonblood donors with caller tunes. ***P<.001.

**Figure 4:**
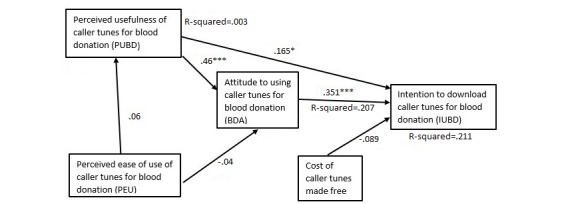
Path model for blood donors with no caller tunes. *P=.019; ***P<.001.

**Figure 5:**
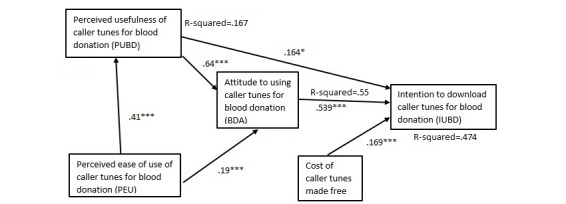
Path model for nonblood donors with no caller tunes. *P=.01; ***P<.001.

We hypothesized that perceived ease of use will have a positive effect on perceived usefulness of caller tunes. The path coefficient was positive for blood donors or nonblood donors with or without caller tunes and statistically significant for all models except that for blood donors with no caller tunes (see Table 8 and Figures 2-5).

We hypothesized that perceived ease of use will have a positive effect on attitudes to caller tunes. All the path coefficients were positive. However, other than nonblood donors with no caller tunes, which was statistically significant (*P*=.001; see Figure 5,Table 8), all the other models had statistically nonsignificant relationship between perceived ease of use and perceived usefulness of caller tunes.

We hypothesized that perceived usefulness will have a positive effect on attitudes to caller tunes. The findings show that all the models supported this hypothesis.

**Table 6:** Means and SDs of the constructs for blood or nonblood donors with or without caller tunes.

Construct	Blood donors with caller tunes (N=278), mean (SD)	Blood donors with no caller tunes (N=200), mean (SD)	Nonblood donors with caller tunes (N=208), mean (SD)	Nonblood donors with no caller tunes (N=270), mean (SD)
Intention to use caller tunes for promoting blood donation	1.08 (0.27)	1.15 (0.35)	1.86 (1.05)	2.07 (1.13)
Perceived ease of use caller tunes for promoting blood donation	1.97 (1.05)	2.19 (0.61)	1.90 (0.75)	2.05 (1.15)
Perceived usefulness for blood donation	1.87 (0.95)	1.90 (0.81)	2.23 (1.06)	2.15 (1.13)
Attitudes to using caller tunes for blood donation	1.66 (0.68)	1.73 (0.66)	1.87 (0.67)	1.90 (0.92)
Free of cost	2.88 (2.17)	2.90 (2.29)	2.34 (1.67)	2.53 (1.88)

**Table 7:** Model fit indices for blood or nonblood donors with or without caller tunes.

Model or Fit Index	Blood donors with caller tunes (n=278)	Blood donors with no caller tunes (n=200)	Nonblood donors with caller tunes (n=208)	Nonblood donors with no caller tunes (n=270)	Recommended value	Reference
NFI^a^	0.95	0.97	0.92	0.97	≥0.95	64
IFI^b^	0.97	1.02	0.94	0.94	≥0.95	64
Tucker–Lewis index	0.90	1.07	0.78	0.96	≥0.95	64
CFI^c^	0.97	1.00	0.94	0.98	≥0.95	64
RMSEA^d^ (90% CI; *P* value)	0.07 (0.00-0.13; .24)	0 (0.00-0.09; .80)	0.11 (0.05-0.18; .046)	0.08 (0.03-0.14; .12)	<0.06	64
Chi square or degree of freedom ratio	2.3	0.6	3.5	2.9	<5.00	65

^a^NFI: normed fit index.

^b^IFI: incremental fit index.

^c^CFI: comparative fit index.

^d^RMSEA: root mean square error of approximation.

**Table 8:** Path models for blood or nonblood donors with or without caller tunes.

Type of participant	Blood donors with caller tunes (n=278)			Blood donors with no caller tunes (n=200)			Nonblood donors with caller tunes (n=208)			Nonblood donors with no caller tunes (n=270)		
Causal path	Estimate	SE	*P* value	Estimate	SE	*P* value	Estimate	SE	*P* value	Estimate	SE	*P* value
PUBD^a^←PEU^b^	0.27	0.05	<.001	0.06	0.09	.41	0.30	0.09	<.001	0.41	0.06	<.001
BDA^c^←PUBD	0.60	0.04	<.001	0.46	0.05	<.001	0.50	0.04	<.001	0.64	0.04	<.001
BDA←PEU	0.08	0.03	.09	−0.04	0.07	.48	0.03	0.06	.70	0.19	0.04	<.001
IUBD^d^←PUBD	0.293	0.02	<.001	0.165	0.031	.02	0.278	0.064	<.001	0.164	0.063	.01
IUBD←BDA	0.056	0.028	.44	0.351	0.038	<.001	0.384	0.101	<.001	0.539	0.077	<.001
IUBD←CFDBD1^e^	−0.049	0.007	.39	−0.089	0.01	.16	0.104	0.035	.067	0.169	0.026	<.001

^a^PUBD: perceived usefulness for blood donation.

^b^PEU: perceived ease of use caller tunes for promoting blood donation.

^c^BDA refers to attitudes to using caller tunes for blood donation.

^d^IUBD: intention to use caller tunes for promoting blood donation.

^e^CFDBD1 implies free of cost.

Our hypothesis that perceived usefulness will have positive effects on intention to use caller tunes among blood donors with or without caller tunes was supported for all the path models (Table 8). Perceived usefulness had significant effect on intention to use caller tunes among blood donors with caller tunes (beta=.293, *P*<.001), blood donors without caller tunes (beta=.165, *P*=.019), nonblood donors with caller tunes (beta=.278, *P*<.001), and nonblood donors without caller tunes (beta=.164, *P*=.01).

Our hypothesis was that attitude will have positive effects on intention to use caller tunes among blood donors or nonblood donors with or without caller tunes. Attitudes had significant effect on intention to use caller tunes among blood donors without caller tunes (beta=.351, *P*<.001), nonblood donors with caller tunes (beta=.384, *P*<.001), nonblood donors without caller tunes (beta=.539, *P*<.001), but a statistically nonsignificant effect with blood donors with caller tunes (beta=.056, *P*=.44).

We hypothesized that making caller tunes free to download will have positive effects on intention to use caller tunes among blood donors or nonblood donors with or without caller tunes. The effect of free of cost caller tunes on the intention to download and use for blood donation was statistically significant (beta=.169, *P*<.001) only in the case of nonblood donors without caller tunes (Figure 5), whereas this path was statistically not significant in the models for blood donors with caller tunes (Figure 2) and without caller tunes (Figure 4), as well as nonblood donors with caller tunes (Figure 3).

Perceived usefulness and attitudes to caller tunes explained 10.7% of the variance in intention to use caller tunes for blood donation among blood donors with caller tunes, with the variance in intention increasing slightly to 11.2% when free to download was added to the model (Table 9).

Among blood donors with no caller tunes, perceived usefulness, attitudes to using caller tunes for blood donation, and free of cost explained 21.1% of the variance in intention. Without free of cost, the variance in intention was 19.7%. The greatest variance in intention was obtained among nonblood donors, with perceived usefulness, attitudes, and free of cost explaining 34.4% and 47.4% of the variances in intention to use caller tunes among those with caller tunes and those without caller tunes, respectively.

Free of cost reduced the variance in intention to download and use caller tunes for blood donation only in the case of nonblood donors with caller tunes (from 34.7%-34.4%), while slightly increasing the variances in intention for blood donors with caller tunes (from 10.7%-11.2%), blood donors without caller tunes (from 19.7%-21.1%), as well as nonblood donors without caller tunes (46.2%-47.4%). Results for the research models without free of cost have not been reported.

Compared with perceived usefulness, attitudes to using caller tunes for blood donation had the stronger effects on intention to use caller tunes for promoting blood donation (Table 9), with the model for nonblood donors without caller tunes having the largest effect (55%), followed by that for blood donors with caller tunes (39.2%).

Moreover, 39.2% of the variance in attitudes to caller tunes for blood donation was explained by perceived ease of use and perceived usefulness of caller tunes for promoting blood donation among blood donors with caller tunes. The greatest effect of perceived ease of use and perceived usefulness on variance in attitudes to using caller tunes for promoting blood donation (55%) was seen among nonblood donors without caller tunes (Table 9).

**Table 9:** Squared multiple correlations for blood or nonblood donors with or without caller tunes.

Original	Blood donors with caller tunes (n=278)	Blood donors with no caller tunes (n=208)	Nonblood donors with caller tunes (n=208)	Nonblood donors with no caller tunes (n=270)
Perceived usefulness for blood donation	0.074	0.003	0.093	0.167
Attitudes to using caller tunes for blood donation	0.392	0.207	0.256	0.55
Intention to use caller tunes for promoting blood donation	0.112	0.211	0.344	0.474

## Discussion

### Principal Findings

Overall, our results provide empirical evidence for factors that could influence the intention of using mobile phone caller tunes for increasing blood donations. The findings mirror studies elsewhere that found significant relationship between perceived usefulness and attitudes to a health information [31,40,41] and intention to use technology [31,33,42]; and significant relationships between perceived ease of use and attitudes to technology [32,34-37] and perceived usefulness of the technology [38-40,42].

Despite considerable research having been conducted on the TAM, especially in the health care sector, our study is innovative for two reasons. It explores primary exogenous TAM variables— perceived ease of use and usefulness—in the context of the utility of mobile phone caller tunes for promoting blood donation. However, although blood donors or nonblood donors may believe that caller tunes are easy to download and could be useful for promoting blood donation, they may not have the resources or money to enable them to download the caller tunes. Our inclusion of free of cost as an additional variable yields useful information for determining whether caller tunes for blood donation should be free of cost to nonblood or blood donors.

With most of the fit indices within the recommended values, there is likelihood of good fit [64]. However, RMSEA value for blood donors with no caller tune was 0, indicating exact model fit to the data [66]. This is a rare finding in surveys and might have research implication for the specified model for blood donors with no caller tunes. With RMSEA *P* value of .802 for this model (Table 7) based on 90% CI, our data appear to have good fit to the model. At samples sizes of at least 200 such as in this study, the use of 90% CI for estimating RMSEA provides comparable results with 95% CI [67]. RMSEA values of 0.05 to 0.08 indicate close fit [68], thus making the model for nonblood donors with caller tunes the only one that appears not to fit the data. This model also had statistically significant RMSEA (*P*=.046) and had NFI, IFI, Tucker Lewis index, and CFI values outside those recommended, suggesting that it may have implications for further research.

This study did not explore actual use of caller tunes for promoting blood donation because caller tunes for promoting blood donation are yet to be created. Nevertheless, our study is a necessary step for designing behavioral interventions using caller tunes to increase the number of first time donors and to encourage existing donors to donate regularly. It was surprising that perceived ease of use and perceived usefulness (Table 9) explained the greatest variance in attitudes to using caller tunes for blood donation among nonblood donors with no caller tunes (55%). However, although perceived usefulness significantly impacted the attitudes to using caller tunes among all four groups of participants, perceived ease of use significantly impacted the attitudes of only nonblood donors with no caller tunes (*P*=.04). This finding may be because nonblood donors with no caller tunes may be unaware of the ease with which they could get caller tunes onto their phones for promoting blood donation. Thus, should the caller tunes on blood donation be created, more efforts or promotion would be needed to positively influence the attitudes of nonblood donors without caller tunes.

This finding suggests that efforts to promote uptake of caller tunes for blood donation should particularly target nonblood donors who do not already use caller tunes. Given that making caller tunes free to download was statistically significant in increasing intention to use caller tunes among only nonblood donors with no caller tunes, intervention designers would need to ensure that caller tunes for promoting blood donation become free. For example, affordability was found to be a statistically significant predictor of intention to use portable coagulometer devices for self-testing of the international normalized ratio among outpatients attending outpatient anticoagulation services [33].

Perceived ease of use, perceived usefulness, and attitudes are all important factors that could determine blood donors’ and nonblood donors’ intentions to use caller tunes to promote blood donation, should blood donation-themed caller tunes be designed. A caller to a mobile phone with a caller tune in Ghana may first hear “If you like this caller tune, press star to download,” reflecting the ease with which caller tunes may be downloaded. The standardized coefficients for the impact of perceived usefulness on attitudes to using caller tunes for all four groups of participants were positive and statistically significant (Table 9). This finding suggests that perceived usefulness is a strong motivator to developing positive attitudes to using caller tunes, which could in turn increase intention to use caller tunes for blood donation. This finding provides practical implications for designing caller tune-based interventions for promoting blood donations. First, should caller tunes for blood donation become available, blood center staff may need to keep reminding both blood donors and nonblood donors about the usefulness of using caller tunes for promoting blood donation. Second, instead of the standard message “If you like this caller tune, press star to download,” if feasible, perhaps mobile telecommunication companies may need to consider customizing such a message. For example, it could be “If you want to save lives with this caller tune on blood donation, press star to download.”

A surprising finding of this study is that making caller tunes free to download appears to have significant positive effect on intention among only nonblood donors with no caller tunes. Conversely, it had negative effect on intention among blood donors, although the effects were nonsignificant. One reason might be that donors were already donating blood and those nondonors were already using caller tunes. Thus, free download did not have a significant impact on these types of participants. Another potential explanation for this finding may be that blood donors may be more willing to promote blood donation by paying the current cost of caller tunes (which is usually about 10 cents per month). For nonblood donors with no caller tunes, cost may be a factor in their current nonuse of caller tunes.

The findings of our study suggest that TAM has a high explanatory power for behavioral intention to use caller tunes—an information system—especially among nonblood donors (Table 9). This is because the variance in behavioral intention in our study was 34.4% for nonblood donors with caller tunes and 47.4% for nonblood donors with no caller tunes compared with those for blood donors with caller tunes (11.2.%) and blood donors with no caller tunes (21.1%).

### Conclusions

This study is innovative because it applies the TAM to mobile phone caller tunes, a technology that is yet to be explored for promoting blood donation. The study also introduces an external factor—free of cost—and finds that it is particularly relevant for nonblood donors with no caller tunes. Our finding that making caller tunes free of cost could significantly increase intention of nonblood donors with no caller tunes to download them offers insight into the use of caller tunes for blood donation. To the best of our knowledge, this is the first study to explore the determinants of intention for downloading caller tunes to promote blood donation.

Thus, this research contributes to TAM in the context of mobile phone caller tunes. Our research models provide theoretical and practical implications for designing caller tunes for blood donation in Ghana and elsewhere. A major strength of our study is that it helps explain relevant variables that influence intentions for downloading caller tunes for those with or without caller tunes.

Our study contributes to information technology or information systems research in at least three ways. We applied TAM in a new context (ie, mobile phone caller tunes for blood donation), which is distinct from prior studies targeting health information systems. Our findings are consistent with those of many studies showing that perceived ease of use and perceived usefulness are significant predictors of behavioral intention [31,33,38,42]. Our study supports others that have showed significant positive relationship between cost and intention to use information technology [33,44]. However, it is important to note that free of cost is a significant factor particularly for those who do not already have the technology (in our case mobile phone caller tunes) and are also not currently adherent to the intended behavior (in our case blood donation). Measures of perceived ease of use were adapted from prior studies and differentiated for those who have or are without the technology under study. Scholars in Ghana and elsewhere could build on them for studying behavioral intention to use mobile phone caller tunes for promoting blood donation.

### Limitations

This study has some limitations. Unlike mobile phone SMS text messages, mobile phone caller tunes for blood donation is relatively new. More research is needed to help generalize the findings in other populations. This is particularly relevant given that this study relied on a convenience sample that excluded participants who were absent at blood donation sites. In addition, we did not differentiate between different types of blood donors such as regular or paid donors. Future research may require a need to identify intention among different types of blood donors to download caller tunes for promoting blood donation. The study also lacks additional variables for explaining intentions for using caller tunes for blood donation. These factors could include social norms. Moreover, the construct for measuring behavioral intention was based on a single-item variable. A study of single-item variables [69] showed that such items are reliable for constructs that are unlikely to be misunderstood by respondents. Future research may need more items for measuring intentions. Furthermore, because our findings were obtained from cross-sectional data, longitudinal studies are needed to help predict intention to use caller tune for blood donation over time. This is because as people gain experience, beliefs or intentions could change.

## Appendix

Multimedia Appendix 1 Operationalization of constructs.

Multimedia Appendix 2 Supplementary questionnaire focusing only on blood donation and caller tunes.

## Abbreviations

ADR: adverse drug reaction
AVE: average variance extracted
BDA: refers to attitudes to using caller tunes for blood donation
CFDBD1: implies free of cost
CFI: comparative fit index
IFI: incremental fit index
IUBD: intention to use caller tunes for promoting blood donation
mHealth: mobile health
NFI: normed fit index
PEU: perceived ease of use caller tunes for promoting blood donation
PUBBD: perceived usefulness for blood donation
RMSEA: root mean square error of approximation
TAM: technology acceptance model
WHO: World Health Organization
