# Supplementary material for: Determinants of Intention to Use Mobile Phone Caller Tunes to Promote Voluntary Blood Donation: Cross-Sectional Study
Source: JMIR Mhealth Uhealth. 2018 May 4;6(5):e117. doi: 10.2196/mhealth.9752 (PMC5960044; doi:10.2196/mhealth.9752)
Supplement: Multimedia Appendix 1 [file mhealth_v6i5e117_app1.pdf]

Multimedia Appendix 1: Operationalization of constructs

Operationalization of constructs

Construct	Item	Item statement	Reference from which item was adapted
Perceived Ease of Use (with caller tune)	PEU1	I found caller tune easy to download	[39,54]
	PEU2	Learning to download caller tune was easy	[31,38]
	PEU3	Using caller tune is easy	[34,55]
	PEU4	Instruction for getting the caller tune is clear and understandable	[31,56]
	PEU5	It is easy to get caller tune from my mobile telecom operator	[39,54]
	PEU6	It is easy for my phone to download a caller tune	[31,56]
	PEU7	It is easy to remember how to download a caller tune	[39]
Perceived Ease of Use (with no caller tune)	PEU1	I think it would be easy to download caller tune	[31,38]
	PEU2	I believe that the instruction for downloading the caller tune would be clear and easy to understand	[31,56]
	PEU3	I think that caller tune is a flexible technology to interact with	[31,40]
Perceived Usefulness of Caller Tunes	PUBD1	Using caller tunes for promoting blood donation would increase voluntary blood donation	[31,38]
	PUBD2	Using caller tunes for promoting blood donation would lead to first time donors becoming repeat blood donors	[31,38]
	PUBD3	Using caller tunes for promoting blood donation would lead to family blood donors becoming repeat blood donors	[31,38]

Attitude to Using Caller Tunes	BDA1	I think it is a good idea to use caller tunes to encourage blood donation	[31,57]
	BDA2	I find it interesting to use caller tunes for making others become blood donors	[57]
	BDA3	I would feel great using caller tunes for making more people become blood donors	[57]
	BDA4	In my opinion, the use of caller tunes will have a positive impact on blood donation	[31,54]
Intention to Use	IUBD	I intend to use caller tunes for promoting blood donation	[54,55]
Free to Download Caller Tunes	CFDBD 1	Mobile telecommunication network makes it free to download	[44]
