# Supplementary material for: Determinants of Intention to Use Mobile Phone Caller Tunes to Promote Voluntary Blood Donation: Cross-Sectional Study
Source: JMIR Mhealth Uhealth. 2018 May 4;6(5):e117. doi: 10.2196/mhealth.9752 (PMC5960044; doi:10.2196/mhealth.9752)
Supplement: Multimedia Appendix 2 [file mhealth_v6i5e117_app2.pdf]

Multimedia Appendix 2: Supplementary questionnaire focusing only on blood donation and caller tunes

1. Have you ever donated blood?
   - ☐ No
   - ☐ Yes

2. Will you be donating blood today?
   - ☐ No
   - ☐ Yes

3. Do you have a mobile phone?
   - ☐ No
   - ☐ Yes

4. Does your mobile phone have a caller tune?
   - ☐ No
   - ☐ Yes

5. What type of caller tune do you have?
   - ☐ Song
   - ☐ Message
   - ☐ Both song and message

**Perceived Ease of Use**	Strongly agree	Agree	Slightly Agree	Neither agree nor disagree	Slightly Disagree	Disagree	Strongly disagree
6. I found caller tune easy to download	1	2	3	4	5	6	7
7. Learning to download caller tune was easy	1	2	3	4	5	6	7
8. Using caller tune is easy	1	2	3	4	5	6	7
9. Instruction for getting the caller tune is clear and understandable	1	2	3	4	5	6	7
10. It is easy to get caller tune from my mobile telecom operator	1	2	3	4	5	6	7
11. It is easy for my phone to download a caller tune	1	2	3	4	5	6	7
12. It is easy to remember how to download a caller tune.	1	2	3	4	5	6	7

**Perceived Ease of Use for respondents with no caller tune**	Strongly agree	Agree	Slightly Agree	Neither agree nor disagree	Slightly Disagree	Disagree	Strongly disagree
13. I think it would be easy to download caller tune	1	2	3	4	5	6	7
14. I believe that the instruction for downloading the caller tune would be clear and easy to understand	1	2	3	4	5	6	7
15. I think that caller tune is a flexible technology to interact with	1	2	3	4	5	6	7

**Perceived Usefulness (blood donation)**	Strongly agree	Agree	Slightly Agree	Neither agree nor disagree	Slightly Disagree	Disagree	Strongly disagree
16. Using caller tunes for promoting blood donation would increase voluntary blood donation	1	2	3	4	5	6	7
17. Using caller tunes for promoting blood donation would lead to first time donors becoming repeat blood donors	1	2	3	4	5	6	7
18. Using caller tunes for promoting blood donation would lead to family blood donors becoming repeat blood donors	1	2	3	4	5	6	7

**Blood donation attitudes**	Strongly agree	Agree	Slightly Agree	Neither agree nor disagree	Slightly Disagree	Disagree	Strongly disagree
19. I think it is a good idea to use caller to encourage blood donation	1	2	3	4	5	6	7
20. I find it interesting to use caller tunes for making others become blood donors	1	2	3	4	5	6	7
21. I would feel great using caller tunes for making more people become blood donors	1	2	3	4	5	6	7
22. In my opinion, the use of caller tunes will have a positive impact on blood donation	1	2	3	4	5	6	7

**Intentions**	Strongly agree	Agree	Slightly Agree	Neither agree nor disagree	Slightly Disagree	Disagree	Strongly disagree
23. I intend to use caller tunes for promoting blood donation	1	2	3	4	5	6	7

24. What are the considerations for designing, your adoption or non-adoption of caller tunes for health?

	Strongly agree	Agree	Slightly Agree	Neither agree nor disagree	Slightly Disagree	Slightly Disagree	Strongly disagree
a. Mobile telecommunication network makes it free to download	1	2	3	4	5	6	7

25. How old are you as of your last birthday?

- ☐ 20 or less
- ☐ 21-30
- ☐ 31-40
- ☐ 41-50
- ☐ 51-60
- ☐ More than 60

26. What is your gender?

- ☐ Male
- ☐ Female

27. What is the highest level of education?

- ☐ Primary
- ☐ Middle school
- ☐ JSS/Junior High School
- ☐ SSS/Senior High School
- ☐ Tertiary educational level
